# Cytotoxicity of intracanal dressings on apical papilla cells differ upon activation with *E. faecalis* LTA

**DOI:** 10.1590/1678-7757-2018-0291

**Published:** 2019-02-21

**Authors:** Carla Renata SIPERT, Aline Pereira OLIVEIRA, Celso Luiz CALDEIRA

**Affiliations:** 1 Universidade de São Paulo, Faculdade de Odontologia, Departamento de Dentística, São Paulo, Brasil.

**Keywords:** Immunity, Cellular, Dental pulp cavity, Cell culture techniques

## Abstract

**Objective:**

The aim of this study was to investigate the cytotoxic effects of modified triple antibiotic paste and an experimental composition using calcium hydroxide on lipoteichoic acid (LTA)-primed apical papilla cells (APC).

**Material and Methods:**

Human APC were tested for *in vitro* cytotoxicity of modified Triple Antibiotic Paste (mTAP – Ciprofloxacin, Metronidazole and Cefaclor at 1:1:1) and of a paste of Ciprofloxacin, Metronidazole and Calcium hydroxide (CMC – 1:1:2) and modified CMC (mCMC – 2:2:1) by using MTT assay. The substances were reconstituted in DMEM at 1,000 µg/mL and ¼ serially diluted before being kept in contact with cells for 1, 3, 5 and 7 days. Further, cells were primed with 1 µg/mL of *Enterococcus faecalis* LTA for 7 days prior to the viability test with 1,000 µg/mL of each substance. Statistical analysis was performed using one-way analysis of variance (ANOVA) and two-way ANOVA respectively followed by Tukey’s post-test. Significance levels were set at p<0.05.

**Results:**

In the first assay, the higher cytotoxic rates were reached by mTAP for all experimental periods. CMC was found toxic for APC at 5 and 7 days, whereas mCMC did not affect the cell viability. Only CMC and mCMC were able to induce some cellular proliferation. In the second assay, when considering the condition with medium only, LTA-primed cells significantly proliferated in comparison to LTA-untreated ones. At this context, mTAP and CMC showed similar cytotoxicity than the observed for LTA-untreated cells, while mCMC was shown cytotoxic at 7 days only for LTA-primed APC. Comparing the medications, mTAP was more cytotoxic than CMC and mCMC.

**Conclusion:**

mTAP showed higher cytotoxicity than CMC and mCMC and the effect of topic antimicrobials might differ when tested against apical papilla cells under physiological or activated conditions.

## Introduction

Regenerative Endodontic Procedures are considered one of the main issues discussed at contemporary Endodontics. Since the protocol stated by Banchs and Trope^1^ (2004), several studies have been published focusing on the treatment of immature permanent teeth regarding the efficacy of clinical procedures and materials to improve the outcome of revascularization protocols. The last one is based on the treatment of the infected pulp cavity with antimicrobials used as intracanal dressings followed by the revascularization of the root canal by bleeding induction and coagulum formation. Differently from the traditional treatment, the options for immature permanent teeth revascularization usually results in thickening of dentinal walls and increased root length, thus leading to a better prognosis than apexification itself.^1^

Considering that microbial reduction in revascularization is obtained by irrigation and intracanal dressing, the choice of substances might be the key factor for effective disinfection of pulp cavity.^1^ Proposed by Hoshino, et al.^2^ (1996), the triple antibiotic paste (TAP) was suggested as an intracanal dressing for revascularization procedures instead of Calcium Hydroxide (CH) due to the induction of tissue necrosis by the latter.^1^ However, *in vitro* studies on apical papilla cells have demonstrated higher cytotoxicity and lower differentiation rates of TAP in comparison to CH under the same concentrations^3^ ^,^ ^4^ or even using TAP at lower concentrations than other substances.^5^ Besides, the lower attachment of the cells to dentin slices treated with TAP in comparison to the CH^6^ was observed. Another important issue to consider is the discoloration resulting from the presence of minocycline in TAP formulations^7^ and therefore its replacement by cefaclor was previously tested by Ruparel, et al.^3^ (2012) with successful clinical outcome.^8^

Based on cytotoxic data regarding TAP paste, CH is now being proposed for revascularization procedures due to its biocompatibility and antimicrobial activity.^7^ However, some microorganisms, such as *Enterococcus faecalis*, are resistant to this substance due to intrinsic mechanisms that lead to bacterial survival instead of the high pH levels resulting from hydroxyl ions release.^9^ Aiming to improve the antimicrobial effect of calcium hydroxide, Pallotta et al.^10^ (2007) proposed its association with ciprofloxacin and metronidazole (CMC). The former inhibits DNA replication and the latter is highly effective against anaerobic bacteria. The authors showed increased antimicrobial effectiveness of CMC against *E. faecalis*, *S. aureus* and *B. fragilis* in comparison to CH alone.^10^

The presence of intracanal infection in teeth with immature root development and necrotic pulps is known since the study by Cvek, et al.^11^ (1976). Bacterial by-products such as lipopolysaccharide (LPS) and lipoteichoic acid (LTA) will be able to activate the residing cells leading to the production of inflammatory mediators.^12^ Among them, the tumor necrosis factor (TNF)-α was demonstrated as able to modulate the differentiation potential of apical papilla cells (APC) *in vitro*.^13^ However, the influence of the previous contact of apical papilla cells with bacterial by-products on the cytotoxicity of substances is an issue that requires special attention considering intracanal irrigating solutions and interappointment medications.

Cultured human APC are being used as a useful methodological tool to investigate the cytotoxic effect of intracanal dressings or sealing cements employed in revascularization protocols.^3^ ^-^ ^6^ ^,^ ^14^ However, *in vitro* studies usually do not consider the activation state of the cells at the time they would clinically be kept in contact with the intracanal medications. To the best of our knowledge, the effect of antimicrobials on APC previously primed with bacterial byproducts is still not investigated.

Considering the importance of survival of apex surrounding cells (including eventually remaining apical papilla cells) after root canal disinfection prior to revascularization procedures, this study aimed to investigate the cytotoxicity of a modified triple antibiotic paste (mTAP) and formulations including ciprofloxacin, metronidazole and calcium hydroxide (CMC and modified CMC) on human cultured apical papilla cells under LTA-untreated or LTA-primed conditions. The null hypothesis is that neither medications (mTAP, CMC or mCMC) or cellular condition (LTA-untreated or LTA-primed) will affect the cellular viability*.*

## Material and methods

### Primary culture of Apical Papilla Cells (APC)

Experiments were conducted in accordance with the Declaration of Helsinki. Ethical approval was obtained from the Ethics Committee for Human Research from the Institution (Process #569.110 CAAE 26843914.0.0000.0075). APC culture was established from the apical papilla of two normal impacted third molars from the same subject with two thirds of the root completed. The teeth were extracted from a female patient (aged 18) for orthodontic reason. The donor signed an informed consent form. The teeth were disinfected and the apical papilla was manually removed from the tooth root. Tissues were minced and then incubated in culture flasks (25 cm^2^) for cell growth in Dulbecco’s modified Eagle’s medium (DMEM) (Invitrogen – Thermo Fisher Scientific, Waltham, MA, USA) with 10% fetal bovine serum (FBS) (Gibco – Thermo Fisher Scientific, Waltham, MA, USA) and antibiotics (100 µg/mL penicillin, 100 µg/mL streptomycin, 0.5 mg/mL amphotericin B – Invitrogen) at standard culture conditions (37°C, 100% humidity, 5% CO_2_ and 95% air). Explants were kept for two weeks under medium replacement every day until migrating cells reached confluence. After the first passage with solution of 0.25% Trypsin/EDTA (Gibco – Thermo Fisher Scientific, Waltham, MA, USA), medium changes occurred every other day. Cells were used between the fourth and eighth passages.^15^ ^,^ ^16^

### Phenotypic characterization of APC

Cultured cells were characterized on the basis of their mesenchymal origin by positivity for vimentin. Cells were fixed in acetone and immunostaining substance with mouse anti-vimentin (Santa Cruz Biotechnology, Dallas, TX, USA) followed by DyLight 594 conjugated anti-mouse IgG (Vector Labs, Burlingame, CA, USA). Slides were then mounted using mounting medium containing DAPI (4’,6-diamidino-2-phenylindole dihydrochloride hydrate) for DNA staining. Images were captured by an inverted confocal microscope (Leica TCS-SPE; Leica, Wetzlar, Hesse, Germany).

### Preparation of antimicrobials

Three intracanal antimicrobial formulations were tested in this study: a modified Triple Antibiotic Paste (mTAP: ciprofloxacin, metronidazole and cefaclor – 1:1:1)^3^ prepared according to recommendations of the American Association of Endodontists and two experimental intracanal dressings containing Ciprofloxacin, Metronidazole and Calcium hydroxide at distinct proportions: 1:1:2 (CMC) and 2:2:1 (modified CMC – mCMC) based on data from Pallotta, et al.^10^ (2007). The medications were individually prepared to achieve precise quantities. All three powdered formulations (Farmácia Fórmula & Ação, São Paulo, SP, Brazil) were individually reconstituted at 1,000 µg/mL in DMEM 10% SBF and incubated at 37°C for 24 h. Solutions were sterilized by filtering at 0.22 µm pore size microfilters (Millipore, Billerica, MA, USA) and serially four-fold diluted (250, 62.5, 15.62 and 3.9 µg/mL) in 10% SBF DMEM.

### Cytotoxicity assay

APC were detached, counted and seeded at 1.25×10^4^ cells/well in 96-well plates. After 24 hours, 100 µL of culture medium alone or containing diluted intracanal dressings were added to the cells in triplicate. After 1, 3, 5 and 7 days, the cell supernatant was replaced by 20 μL of a solution of 5 mg/mL of MTT [3-(4,5-Dimethylthiazol-2-yl)-2,5-diphenyltetrazolium bromide] (Sigma-Aldrich, St. Louis, MO, USA) in phosphate buffered saline (Merck, Darmstadt, Hessen, Germany), followed by 180 μL of 10% FBS DMEM. Cells were incubated for 4 hours and MTT solution was replaced by 100 µL of isopropanol. Optical density was determined using a plate reader (Synergy HT, Biotek, Instruments, Inc. Winooski, VT, USA) at the wavelength of 570 nm. Experiments were conducted in triplicate.

### Priming of the APC with *Enterococcus faecalis* LTA

**Figure 1:**
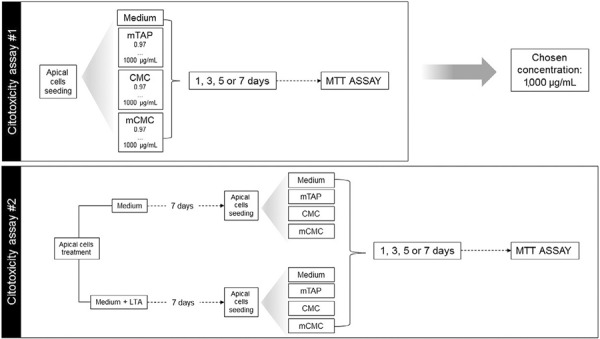
Experimental design for the cytotoxicity study of apical papilla cells

To understand the effect of innate immunity activation on cytotoxic effect of intracanal dressings on ACP; part of the cells were primed with 1 µg/mL LTA from *E. faecalis* (L4015, Sigma-Aldrich, St. Louis, MO, USA) for 7 days with medium change every other day.^17^ Next, cells were detached, counted and seeded as described above. After 24 h, medium only or containing medications at 1,000 µg/mL was added to the wells and the Untreated- or LTA-primed-APC viability was assessed after 1, 3, 5 and 7 days. Experiments were conducted in triplicate. The experimental design is summarized at Figure 1.

### Statistical analysis

Statistical analysis was performed using GraphPad Prism 5.0 (GraphPad Software, San Diego, CA, USA). Data resulting from the first set of experiments were subjected to one-way analysis of variance (ANOVA) followed by Tukey’s post-test for comparison between pairs of groups. Data from the second experiment were analyzed using two-way ANOVA followed by Tukey’s post-test. Significance level was set at p<0.05.

## Results

### Characterization

**Figure 2:**
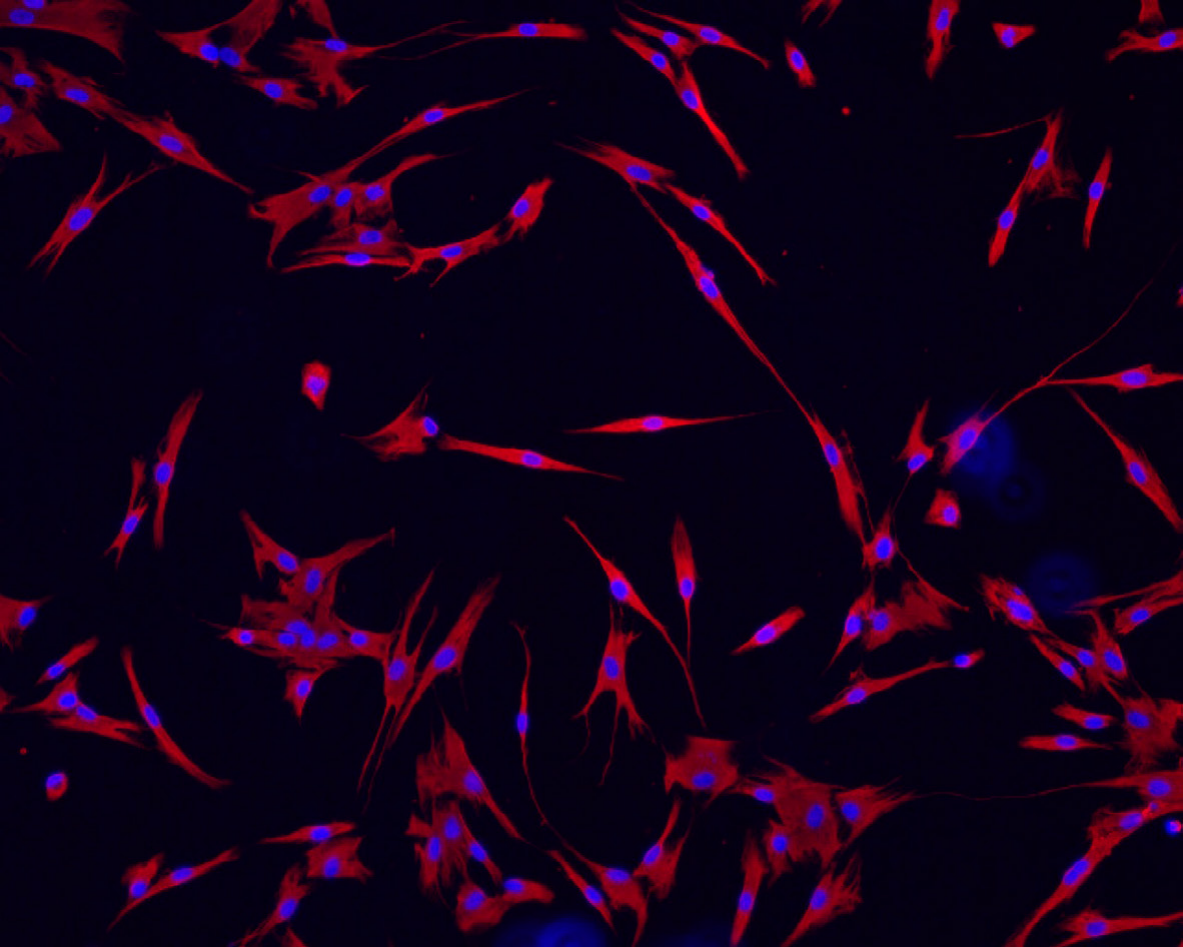
Characterization of human cultured apical papilla cells (APC) by vimentin. Cultured APC were immunostained for vimentin. Image captured by a fluorescence microscope at 40×

The immunostaining for vimentin was observed for the whole cellular population, thus confirming the mesenchymal origin of human APC culture (Figure 2).

### Cytotoxicity assay

**Figure 3:**
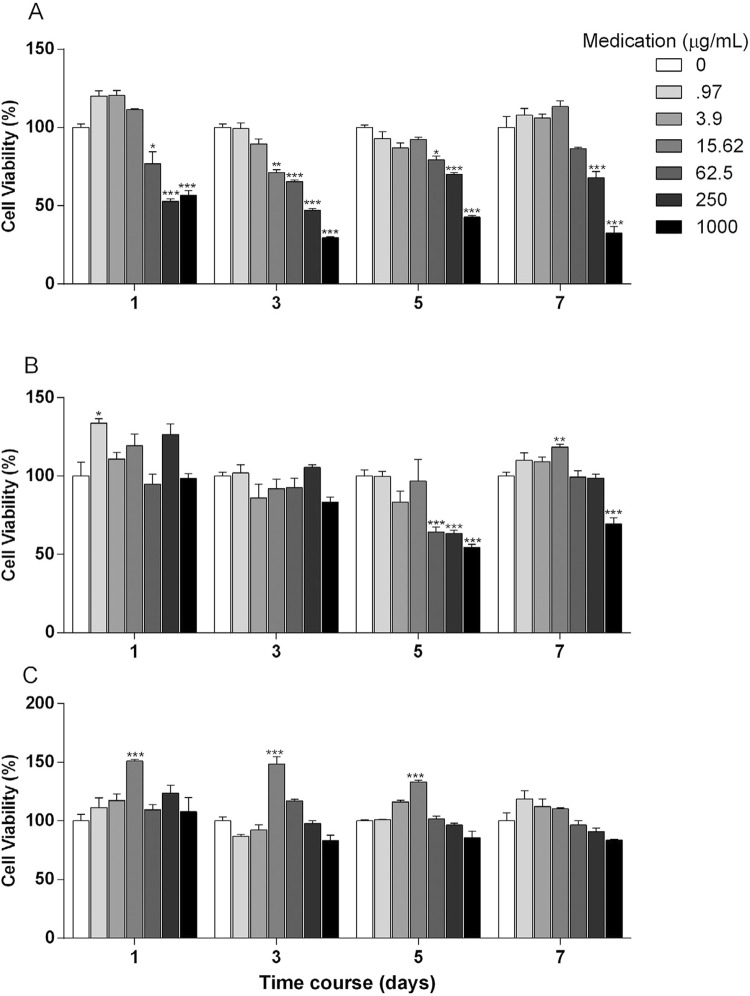
Cytotoxicity of antimicrobials on APC. *In vitro* cytotoxicity of mTAP (A), CMC (B) and mCMC (C) at indicated concentrations in contact with APC for 1, 3, 5 and 7 days. Cell viability detected by MTT assay and normalized based on control (untreated) cells. *p<.05, **p<.01, and ***p<.001 in comparison to culture medium alone (0) (n=3)

Results for cytotoxicity assay #1 are shown in Figure 3. The mTAP showed increased cytotoxic effect in a concentration-response relationship for all the tested experimental periods (Figure 3A). Depending on the concentration tested, CMC-treated cells presented viability loss at days 5 and 7 and increase in metabolic activity at days 1 and 7 (Figure 3B). Cells kept in contact with mCMC showed no significant viability loss at the experimental periods tested, but showed increased MTT metabolization at days 1, 3 and 5 (Figure 3C), which might be suggested as cell proliferation.

**Figure 4:**
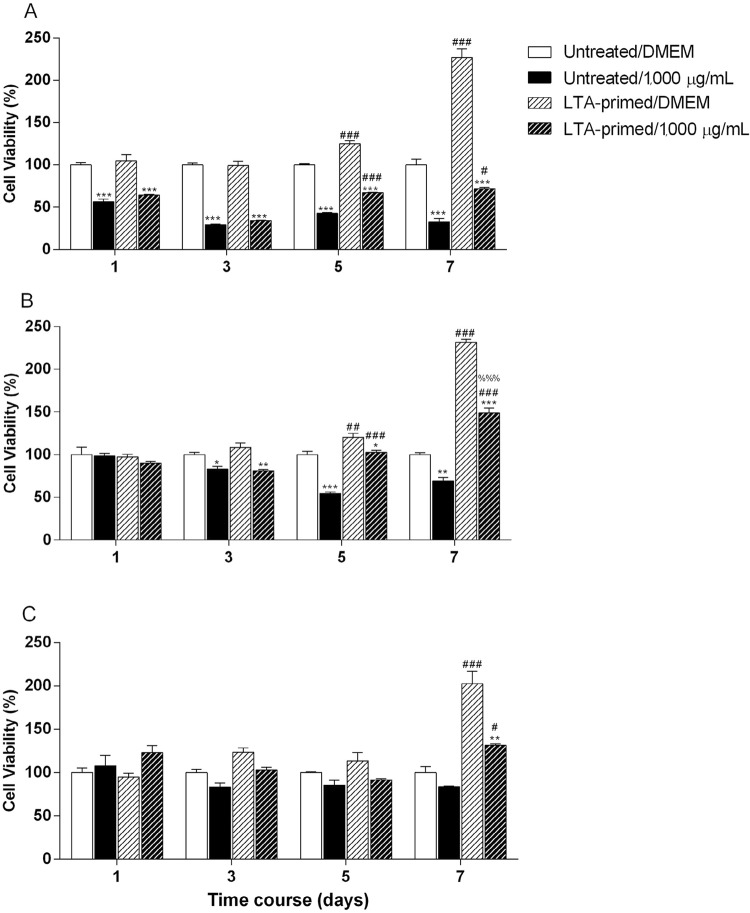
Cytotoxicity of antimicrobials on Untreated and LTA-primed APC. In vitro cytotoxicity of mTAP (A), CMC (B) and mCMC (C) at 1,000 µg/mL tested in contact with untreated or LTA-primed APC for 1, 3, 5 and 7 days. Cell viability detected by MTT and normalized based on control (Untreated/DMEM) cells. *p<.05, **p<.01 and ***p<.001 in comparison to the respective cell condition with culture medium alone (0) or # in comparison to the respective stimuli on untreated cells and % compared to untreated cells under medium alone (n=3)

Data resulting from cytotoxicity assay #2 are shown in Figure 4. The mTAP was shown cytotoxic at all the time periods tested for untreated and LTA-primed cells. The pretreatment with LTA increased the cell proliferation at days 5 and 7 (Figure 4A). The cytotoxicity of CMC on APC was observed at days 3, 5 and 7 also for both untreated and LTA-primed cells (Figure 4B). Finally, the cytotoxicity of mCMC, not observed for LTA-untreated cells, was shown for LTA-primed ones at day 7 as a result of cell proliferation induced by LTA when compared to the respective control (LTA-primed/DMEM) (Figure 4C).

**Figure 5:**
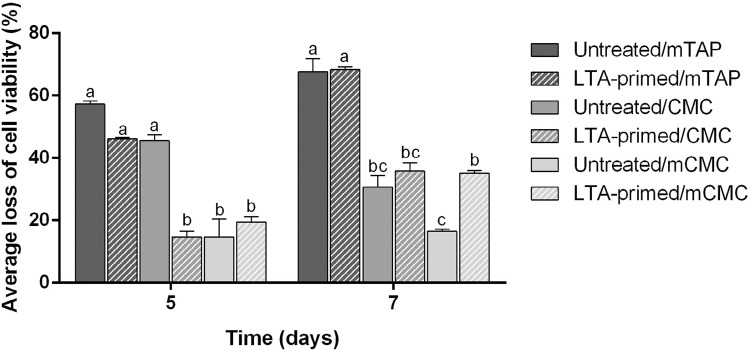
Average loss of cell viability for APC. Data regarding the viability of the tested medications were normalized based on their respective control (Untreated or LTA-primed) for calculation of the percentage of loss of cell viability. Different letters mean statistical significance (p<0.05) (n=3)

A statistically significant interaction between "medication" and "cellular activation" was found for CMC at day 5 (p=0.0029) and for the three medications tested at day 7 for mTAP (p=0.0002), for CMC (p=0.0002) and for mCMC (p=0.0102) by two-way ANOVA. Based on this result, for days 5 and 7, an analysis of the percentage of viability loss was performed after normalizing the datum of each medication sample with their respective control (untreated or LTA-primed cells). This data were analyzed by one way ANOVA and the result is shown at Figure 5. At day 5, mTAP was significantly more cytotoxic than CMC for LTA-primed cells and mCMC for both cellular conditions. CMC was more cytotoxic than mCMC only compared to untreated cells. At day 7, mTAP reached the higher cytotoxicity levels with more than 60% of viability loss. CMC was less cytotoxic than mTAP for both cellular conditions and similar to mCMC. However, mCMC cytotoxicity was significantly increased when considering LTA-primed cells compared to untreated ones (Figure 5).

## Discussion

The endodontic treatment of immature teeth due to caries or trauma has been revisited since the dissemination of regenerative endodontic procedures,^1^ which nowadays are considered one of the scopes of Contemporary Endodontics (AAE Position Statement). In this context, important studies were carried out to evaluate the antimicrobial efficacy and the biocompatibility of substances employed as intracanal dressings for regenerative endodontics.^3^ ^,^ ^4^ ^,^ ^6^ ^,^ ^14^ ^,^ ^18^ Considering the eventual need for apical papilla cells survival to promote hard tissue deposition, this study aimed to investigate the cytotoxicity of the modified triple antibiotic paste (mTAP) and other experimental formulations including ciprofloxacin, metronidazole and calcium hydroxide (CMC and mCMC) over untreated or LTA-primed apical papilla cells. We demonstrated that the presence of bacterial by-products and intracanal medications alter the metabolic behavior of apical papilla cells.

Previous *in vitro* studies investigated the cytotoxicity of intracanal medications and endodontic cements employed in regenerative procedures over apical papilla cells.^3^ ^,^ ^4^ ^,^ ^14^ ^,^ ^18^ However, the interruption of root formation as a consequence of caries or trauma is usually accompanied by microbial colonization of the pulp cavity^19^ even in the presence of vital remnants of apical papilla or periodontal ligament cells.^20^ In this context, Böttcher, et al.^21^ (2013) demonstrated the loss of immunostaining for stromatin-1 and bone morphogenetic protein-4 at the apical papilla of rats subjected to experimentally-induced root formation interruption. These data strongly suggest that vital remnants of apical papilla eventually found at the root apices of non-vital immature teeth might be phenotypically altered at the time of endodontics intervention. One may also speculate that the physiological function of these remaining cells could be compromised. To the best of our knowledge, the effect of cellular activation by bacterial by-products on the cytotoxicity of chemical substances used as intracanal medications has not been previously assessed. In this study, APC were previously primed with LTA to establish a low virulence microenvironment based on the cellular activation by a Gram-positive bacteria by-product^17^ ^,^ ^22^ commonly found in endodontic infections^23^ and highly prevalent in necrotic immature teeth.^19^

In this study, mTAP demonstrated an important viability decrease (over 50%) with 250 and 1,000 µg/mL at 1 day of culture. At day 3, cytotoxic effect was observed at the concentrations ranging from 15.62 to 1,000 µg/mL. Interestingly, at days 5 and 7 the cytotoxicity was seen *de novo* with 250 and 1,000 µg/mL (Figure 2A). These data contrast with the findings by Ruparel, et al.^3^ (2012) that did not find toxic effect of mTAP at 100 µg/mL. Considering the original formulation with minocycline, Chuensombat, et al.^18^ (2013) showed about 50% of viability reduction for dental pulp cells and almost 70% for apical papilla cells in contact with 25 µg/mL of TAP (3Mix) for 7 days. Phumpatrakom and Srisuwan^4^ (2014) reported complete cell death with TAP at 1 mg/mL at 7 days of culture. Kitikuson and Srisuwan^6^ (2016) demonstrated that the pre-treatment of dentin with TAP compromised the APC attachment to root surfaces. Recently, TAP was shown cytotoxic for APC at 50 µg/mL, but not at 10 µg/mL at 3 and 5 days.^5^

Experimental formulations containing ciprofloxacin, metronidazole and calcium hydroxide were tested in this study at two distinct powder proportions: 1:1:2 (CMC) and 2:2:1 (mCMC). The study of Pallotta, et al.^10^ (2007) demonstrated that CMC presented 125 µg/mL as minimum inhibitory concentration (MIC) for *E. faecalis*, *P. aeruginosa* and *B. fragilis* and 500 µg/mL for *S. aureus*. The same study presented 16 mg/mL as MIC of calcium hydroxide for the first three microorganisms while no inhibitory action was observed for *S. aureus.* Considering these data, it seems clear that the addition of ciprofloxacin and metronidazole to calcium hydroxide increases significantly the antibacterial effect of the intracanal dressing. Considering together the results of Pallotta, et al.^10^ (2007) and our results of cytotoxicity of CMC, we experimentally tested a proportion that uses the twice amount of ciprofloxacin and metronidazole and the half one of calcium hydroxide. The last one is well known by its antimicrobial activity and biocompatibility on APC survival and attachment and was found to induce cellular proliferation at the same concentration that other substances demonstrated significant toxicity.^3^ ^,^ ^6^ ^,^ ^24^ We could speculate that the addition of calcium hydroxide replacing cefaclor in the mTAP may result in a pH balance besides the improvement in the antimicrobial effect of the intracanal dressing. These two formulations were tested over apical papilla cells showing less cytotoxicity than mTAP (Figure 5). Decrease in cell viability was shown by CMC at days 5 and 7 and no effect by mCMC at all the experimental periods tested. Interestingly, significant increase in cell viability was observed for the concentration of 15.62 µg/mL at days of culture 1, 3 and 5. These data corroborate the findings of Ruparel, et al.^3^ (2012) regarding the ability of calcium hydroxide to induce cellular proliferation *in vitro*. The cytotoxic effect of substances used as intracanal dressing depends on concentration and combination of antimicrobials. This is clearly stated by Chuensombat, et al.^17^ (2013), who showed higher cytotoxicity for 3Mix than the drugs tested separately at the same concentrations. Similar behavior is observed at APC when subjected to CMC and mCMC at this study, since the latter presents more antibiotics than the former. We may speculate that a possible balance among the concentration of the three substances might differentially affect cell survival. Nevertheless, the mechanisms involved in the distinct cytotoxic rates observed for the same substances used at distinct proportions must be further investigated.

The previous studies of the literature regarding the cytotoxic effect of intracanal substances over APC directly or for surfaces treatment investigated the influence of the chemicals on cells under physiological conditions, i.e., without any previous activation. Our findings demonstrated that priming cells with *E. faecalis* LTA previously to the cytotoxicity assay resulted in significant increase of viability, thus strongly suggesting the cellular proliferation at this context. This was consistently observed for the latter experimental periods (5 and 7 days). Especially considering the experimental medicaments (CMC and mCMC), the amount of remaining LTA-primed cells in contact with the substances was higher than the untreated ones. More importantly, on the mCMC group the cytotoxic effect of the substance was only seen at the LTA-primed cells group (LTA-primed/1000 µg) with significant decrease in cell viability compared to the respective LTA-primed untreated control (LTA-primed/DMEM) (Figure 4C). Statistical analysis showed positive interaction between medication and state of cellular activation, which suggested that the toxic effect of substances might depend on the previous condition of the cells/tissue treated. These data highlight the importance of considering the context of cellular microenvironment for dental materials cytotoxicity tests. Materials such as mCMC could be considered highly biocompatible, but the same might not be true if cells are previously subjected to bacterial byproducts activation. These findings allow us to suggest that the presence of microbial molecules remnants might significantly influence the tissue response to dental materials.

The increase in LTA-primed cell viability observed from our data might be suggested as cellular proliferation or metabolism augmentation. Conclusions regarding biocompatibility based on this finding must be carefully considered since we did not investigate the cellular function in this study. On the other hand, the basal metabolic condition of APC might be considered at cytotoxicity tests since these cells are under a pro-inflammatory microenvironment at the time of clinical intervention. Future studies are needed to elucidate the effect of APC activation on the molecular modulation resulting from endodontic materials since their effect investigated only on healthy microenvironments might lead to overestimated conclusions regarding their biocompatibility. In conclusion, mTAP and CMC were found cytotoxic in a concentration and time-dependent manner for LTA-untreated cells while mCMC did not show cytotoxicity at this condition. Under activation by LTA, the mTAP, CMC and mCMC were found cytotoxic for LTA- primed cells by inhibiting the proliferation induced in response to the bacterial byproduct contact. Moreover, the cytotoxicity of topic antimicrobials on apical papilla cells differ under physiological or activated conditions. Considering our findings, the null hypothesis of this study was rejected.

## Conclusion

The mTAP had the highest cytotoxicity compared to CMC and mCMC on apical papilla cells. The last one, in turn, was cytotoxic only for LTA-primed cells. Moreover, at viability tests *in vitro*, the cytotoxic effect of intracanal dressings might depend on the condition of cellular activation by bacterial byproducts*.*
